# Tracing the path of carbon export in the ocean though DNA sequencing of individual sinking particles

**DOI:** 10.1038/s41396-022-01239-2

**Published:** 2022-04-20

**Authors:** Colleen A. Durkin, Ivona Cetinić, Margaret Estapa, Zrinka Ljubešić, Maja Mucko, Aimee Neeley, Melissa Omand

**Affiliations:** 1 Monterey Bay Aquarium Research Institute, Moss Landing, CA USA; 2 Universities Space Research Association, Columbia, MD USA; 3 Ocean Ecology Laboratory, NASA/Goddard Space Flight Center, Greenbelt, MD USA; 4 School of Marine Sciences, Darling Marine Center, University of Maine, Walpole, ME USA; 5 Biology Department, Faculty of Science, University of Zagreb, Zagreb, Croatia; 6 Science Systems and Applications, Inc., Lanham, MD USA; 7 Graduate School of Oceanography, University of Rhode Island, Narragansett, RI USA; 8 Present Address: GESTAR II, Morgan State University, Baltimore, MD USA

**Keywords:** Biogeochemistry, Microbial biooceanography

## Abstract

Surface phytoplankton communities were linked with the carbon they export into the deep ocean by comparing 18 S rRNA gene sequence communities from surface seawater and individually isolated sinking particles. Particles were collected in sediment traps deployed at locations in the North Pacific subtropical gyre and the California Current. DNA was isolated from individual particles, bulk-collected trap particles, and the surface seawater. The relative sequence abundance of exported phytoplankton taxa in the surface water varied across functional groups and ecosystems. Of the sequences detected in sinking particles, about half were present in large (>300 μm), individually isolated particles and primarily belonged to taxa with small cell sizes (<50 µm). Exported phytoplankton taxa detected only in bulk trap samples, and thus presumably packaged in the smaller sinking size fraction, contained taxa that typically have large cell sizes (>500 μm). The effect of particle degradation on the detectable 18 S rRNA gene community differed across taxa, and differences in community composition among individual particles from the same location largely reflected differences in relative degradation state. Using these data and particle imaging, we present an approach that incorporates genetic diversity into mechanistic models of the ocean’s biological carbon pump, which will lead to better quantification of the ocean’s carbon cycle.

## Introduction

Phytoplankton living in the surface ocean are responsible for half of the primary production on Earth each year [1]. A fraction of that carbon is exported into the ocean’s interior, where it can remain sequestered away from the atmosphere for hundreds to thousands of years, or for geological time scales if buried in the sediments [2]. The biological, chemical, and physical processes that result in particulate organic carbon (POC) transport to depth are collectively referred to as the biological carbon pump [2, 3]. The strength and efficiency of the biological carbon pump is poorly constrained, in part because we lack a mechanistic understanding of which primary producers are exported and the key ecological conditions required for export to occur [4–6]. More accurate models of the ocean’s carbon cycle will need to incorporate observations that resolve the ecological mechanisms of carbon transfer [4].

Technological advances now enable observations of plankton communities to be collected at high taxonomic resolution and at global scales. These technologies include ocean color satellites, such as NASA’s upcoming Plankton, Aerosol, Cloud, ocean Ecosystem (PACE) mission [7], in situ imaging technologies [8, 9], and high-throughput DNA sequencing approaches [10, 11]. While observations of the surface plankton community are steadily expanding, there is currently no mechanistic framework that enables these measures of biological diversity to be integrated into models of the biological carbon pump.

Recent studies have explored the link between phytoplankton communities and POC export into the deep ocean by sequencing the nucleic acids associated with sediment trap-collected particles [12–17]. Though the phytoplankton contents of sinking particles have long been examined visually (for example [18–21], among many), molecular approaches are advantageous because they can be high-throughput and are sensitive enough to detect organisms that are fragmented or difficult to visually identify. In many cases, molecular-based studies verify the results of more painstaking microscopy-based studies. For example, both approaches have found that pico- and nanophytoplankton are important export producers [13, 18, 22, 23], that heterotrophic protists can comprise a large proportion of total POC flux [14, 16, 18], and that specific organisms can generate episodic pulses of POC to the seafloor [15, 17, 21, 24–27]. However, no study has integrated phytoplankton molecular data into models of the biological pump because taxa are not yet quantitatively linked to mechanisms of POC flux.

We recently described how microscopy of individually resolved particles collected in sediment trap gel layers can be used to quantify the contribution of different particle types to POC flux [28]. Here, we combined these quantitative, ecologically resolved observations of POC flux with analysis of the DNA sequence communities within individually isolated particles to 1) identify the subset of the surface phytoplankton taxa present within sinking POC and 2) link those taxa with their ecological mechanisms of export and the quantity of carbon exported by each mechanism.

## Methods

### Cruise and sampling platforms

**Fig. 1:**
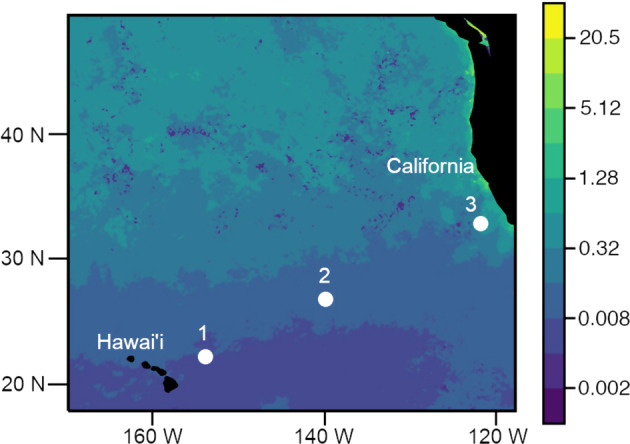
Drifting sediment traps were deployed at three locations (white dots) between Hawai’i and the coast of California, USA. Ocean color indicates February 2017 monthly composite chlorophyll concentrations (mg m^−3^) observed by the MODIS Aqua satellite [54]. Black areas indicate location of Hawai’i and North America.

Samples were collected in boreal winter between Honolulu, Hawai’i and Portland, Oregon aboard the R/V Falkor from 24 January to 20 February 2017 (FK170124). Sediment traps were deployed at 3 locations along the transect, including stations 1 and 2 located in oligotrophic waters and station 3 located in the California Current (Table 1, Fig. 1). Details of trap deployments are reported elsewhere [28]. Briefly, all trap collection tubes were deployed to 150 m for between 1 and 3 days. Trap platforms included a neutrally buoyant sediment trap (NBST) [29] and a surface-tethered Wirewalker (WW) [30] modified to carry a trap frame (KC Denmark) suspended by a bungee below the profiling portion of the array. At Station 3 the WW trap was deployed twice, for two consecutive periods, 1.67 days followed by 1.04 days. The traps were 20 m below the mixed layer depth at Stations 1 and 2, and 110 m below the mixed layer depth at Station 3. Particles collect at different depths below the mixed layer likely undergo different degrees of degradation prior to collection in the sediment traps.

**Table 1:** Summary of sediment trap deployments at each station and the type of sinking particles collected for DNA sequencing. Numbers in parentheses indicate additional particles sampled 24 or 48 h after sample recovery.

Station	Location	Deployment dates	Trap type	Depth (m)	Duration (days)	Bulk particle collection	Number of particles sampled				
							Aggregates	Dense detritus	Long fecal pellets	Large loose fecal pellets	Total
1	22.3°N, 151.9°W	28 Jan−2 Feb 2017	NBST	150	2.88	None	3	7	2	0	**12**
2	27.7°N, 139.5°W	5–8 Feb 2017	WWtrap	150	3.06	Unpreserved and preserved	0	3	2	1	**6**
3	34.7°N, 123.5°W	12–14 Feb 2017	NBST	150	2.60	None	–	–	–	–	–
		12–13 Feb 2017	WWtrap	150	1.67	Unpreserved and preserved	7 (5)	3 (4)	1 (2)	4 (4)	**15 (15)**
		13–14 Feb 2017	WWtrap	150	1.04	Unpreserved and preserved	5 (3)	5 (4)	2 (0)	3 (5)	**15 (12)**

### Sediment trap sampling

Durkin et al. [28] described in detail the preparation of collection tubes, analyses of bulk chemical fluxes, imaging of particles collected in polyacrylamide gel layers, and modeling of POC flux by distinct particle classes based on imagery. Here, we focus on the collection and isolation of nucleic acids from sinking particles. All sediment traps carried 4 collection tubes. One tube on both the NBST (12.7 cm diameter, 70 cm heigh) and WW trap (7 cm diameter, 45 cm heigh, no baffle) contained 0.2 μm filtered seawater overlying a polyacrylamide gel layer [31] for particle imaging and the isolation of individual particles. Two of the remaining three WW trap tubes were filled with 0.2 μm filtered seawater with salinity adjusted to 45, and a bottom layer of either unpreserved brine (500 mL, salinity = 70, 1 tube) or custom made RNAlater-like preservative [32]. At station 1, WW collection tubes were destroyed by a shark attack and individual particles were instead isolated from the gel layer carried by the NBST with no bulk particles available for nucleic acid sampling.

**Fig. 2:**
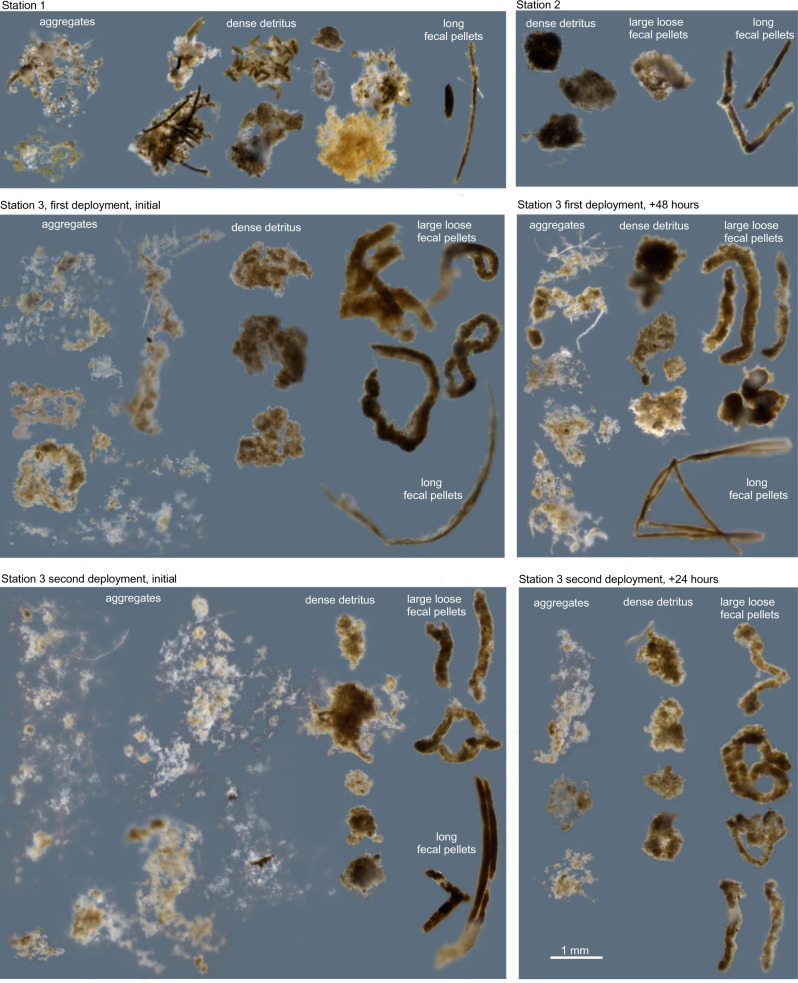
Micrographs of individual particles isolated from gel layers at each station. Particles are arranged by category in columns. Micrograph images were altered by manually removing background regions and adjusting brightness and contrast.

Upon recovery, all trap tubes were allowed to settle on the deck of the ship for an additional hour before overlying seawater was siphoned off. Gel jars were removed from the tube bottoms and seawater remaining in the jar was gently pipetted off the gel surface. Particles collected within the gel sample were imaged using a stereomicroscope (SZX16, Olympus, Tokyo, Japan) with a camera attachment (Infinity 2, Teledyne Lumenera, Ottawa, Canada). Individual particles larger than 300 µm were removed from the gel using a p1000 pipettor by first back-filling the pipette tip with approximately 200–300 μL of nuclease-free water and then continuing to aspirate the selected particle from the gel layer. The sample within the pipette tip was then transferred into a cryovial, with the backfilled water aiding in blow out of viscous gel residue surrounding the particle. Cryovials were flash frozen in liquid nitrogen and stored at −80 °C. Four different particle classes were isolated from the gels (Fig. 2, Table 1). Aggregates were semi-translucent particles with fluffy edges. Dense detritus particles were non-translucent aggregates that often resembled fragmented fecal pellets. Long, cylindrical fecal pellets include those typically produced by crustaceous zooplankton encased in a smooth chitin membrane. Large, loose fecal pellets were likely a mixture of long pellets partially degraded by microorganisms and pellets produced by non-crustacean zooplankton such as pteropods or pyrosomes [28].

At all stations, particles were isolated from the gel layers <24 h after trap recovery, though particles that settled into the unpreserved gel layer continued to degrade while traps were deployed and prior to the time of isolation. To determine how sampling delay time affected the detected 18 S rRNA gene community composition, gel layers collected at Station 3 were incubated at 4 °C for an additional 48 h after the first WW trap deployment and 24 h after second WW trap deployment before additional particles were isolated from the gel layer.

To sample nucleic acids from bulk particle collections, unpreserved brine or RNAlater was drained from the bottom of the collection tubes. Particles were collected by vacuum filtration onto a 0.2 μm polycarbonate filter, placed in cryovials, flash frozen in liquid nitrogen, and stored at −80 °C. Zooplankton were not picked out of these samples prior to processing. Since most zooplankton in sediment traps are likely “swimmers” that actively entered the trap, the nucleic acid sequence community extracted from these bulk samples included actively swimming zooplankton in addition to the passively sinking community.

### Surface seawater sampling

Seawater from the surface mixed layer was collected in Niskin bottles during CTD casts at all three stations during the deployment period of the WW and NBST. Water was collected from just below the surface, at the depth of the chlorophyll maximum, and at the bottom of the mixed layer as defined by the depth of the pycnocline (Supplementary Table 1, Supplementary Fig. 1). Pigment concentrations were measured in surface water samples using high-performance liquid chromatography (HPLC), providing a proxy for the relative biomass of various phytoplankton functional groups [33]. Water samples were also used to extract DNA from the planktonic communities for 18 S rRNA gene sequencing (additional details in Supplementary methods).

### DNA extraction and 18 S rRNA gene amplification

DNA was extracted from individually isolated particles (*n* = 83) by transferring thawed particles into a 5–10% solution of Chelex 100 resin (Hercules, CA, BioRad, USA) in nuclease-free water (0.1 g per mL). Chelex-particle solutions were disrupted by vortexing for 2 min and incubated in a water bath at 95 °C for 10 min, vortexing again for 2 min, incubating at 95 °C for an additional 10 min, and vortexing a final time for 2 min. Chelex and other solid contents were concentrated to the bottom of the sample tube by centrifugation and then the overlying liquid containing nucleic acids was removed. DNA within this liquid layer was further purified and concentrated using the Genomic DNA Clean and Concentrator-10 (Zymo Research, Irvine, CA, USA) with a final elution volume of 50 μL. DNA was extracted from filters containing bulk sediment trap particles (*n* = 6; unpreserved brine or RNAlater) with the PowerViral DNA/RNA extraction kit (Qiagen, Hilden, Germany). DNA from surface seawater samples (*n* = 40) was extracted with phenol:chlorophorm:isoamyl alcohol solvents as previously described [34].

Approximately 420 bp of the V4 region of 18 S rRNA gene sequences were amplified by PCR of DNA extracted from trap-collected particles and seawater samples, as described in Supplementary File 1 (Forward PCR primer 5′-CCAGCASCYGCGGTAATTCC-3′; Reverse PCR primer 5′-ACTTTCGTTCTTGATYRATGA − 3′ [35, 36]). A total of 81 purified, barcoded PCR amplifications from individual particles and bulk sediment trap samples were combined in equal concentration and sequenced at the Michigan State University Genomics Core Facility using an MiSeq v2 (Illumina) Standard flow cell in a 2 × 250 bp paired end format. DNA extracted from seawater samples were sent for 18 S rRNA gene library preparation and MiSeq (Illumina) amplicon sequencing to Molecular Research MrDNA (www.mrdnalab.com, Shallowater, TX, USA). Sequencing was performed in a 2 × 300 bp paired end format. All sequence data are available from the National Center for Biotechnology Information database under accession number PRJNA494700.

### Analysis of DNA sequences

Initial processing of 18 S rRNA gene sequence files was performed using QIIME2 [37] and the DADA2 [38] workflow (Supplementary File 1, 10.5281/zenodo.6383344). Amplicon sequence variants (ASV) were associated with taxonomic identities by comparison to the PR2 database [39] (v 4.12.0, downloaded 24 January 2020) using a naive Bayes classifier [40]. The feature table of read counts and the taxonomy table associating those ASVs with identities was exported from QIIME2 and converted to comma-separated-value files for analysis using Python. ASV taxa were classified as photosynthetic (Supplementary Table 2) versus heterotrophic. All Dinoflagellata (PR2 taxonomy level 3) that were not Syndiniales (PR2 taxonomy level 4) were categorized as phytoplankton due to the variability and plasticity in trophic strategies within this group of organisms [41]. Consequently, dinoflagellate sequences included in phytoplankton-specific analyses likely contained some heterotrophic taxa. Bacillariophyta (i.e. diatoms) were assessed separately from other Ochrophyta taxa due to their distinct ecological function and high diversity. Phytoplankton ASVs that were unique to a sample type (individual particle, bulk trap particles, seawater) versus shared across sample types were assessed to identify different export mechanisms. Sequence communities from the same sample type (bulk trap, individual particle, seawater) were pooled together for these analyses. We assessed how methodological biases affected comparison across different sample types by simulating the effect of changing sample size (see Supplementary methods, Supplementary Figs. 2 and 3). Station 1 samples were not included in this analysis because no bulk sediment trap particle DNA samples were available. The fraction of surface phytoplankton taxa contributing to export was defined as the subset of phytoplankton ASVs detected in seawater samples that were also detected in any individually isolated particle or in bulk collected trap samples. The number of individually isolated particles collected at both stations 2 and 3 was large enough to support this comparison (Supplementary methods, Supplementary Fig. 3c). Heterotroph export from the surface was not assessed in this way because their presence in the particles could also be caused by colonization and growth in the mesopelagic. The particle size in which phytoplankton and heterotrophic taxa were packaged was assessed by identifying the subset of ASVs detected in bulk particle trap samples that were also detected in any individually isolated particle. ASVs detected in the bulk particles but not in the individually isolated particles were assumed to have been packaged within particles that were not selected for isolation, including <300 µm sized particles, zooplankton swimmers, and individual cells. This analysis was only performed at Station 3, because the large number of particles isolated from this location enabled a robust comparison (Supplementary Fig. 3d). To identify whether different particle types exported distinct eukaryotic communities, the composition of relative reads (i.e. percent read abundance) and diversity of total 18 S rRNA gene sequences were compared across all samples. The read counts of each sample were rarified to 31 993 reads across all samples using tools in QIIME2 (qiime diversity core-metrics), eliminating one seawater sample from this analysis (>5 µm composite sample collected at Station 2 on 7 February). The Bray-Curtis dissimilarities were calculated among samples from the rarified ASV feature table using functions in Python’s scipy. Differences in sample groups were assessed by PERMANOVA and Bonferroni *p*-value adjustment for multiple comparisons. To assess differences in ASV richness among samples rather than relative read counts, the non-rarified feature table of ASVs detected at least 10 times across the entire dataset was transformed into a presence/absence table. Uncertainty in results due to stochastic particle collection by sediment traps was constrained by additionally performing separate comparisons of the trap communities collected from the two trap deployments at Station 3.

## Results

### Fraction of surface phytoplankton nucleic acids in sinking particles

**Fig. 3:**
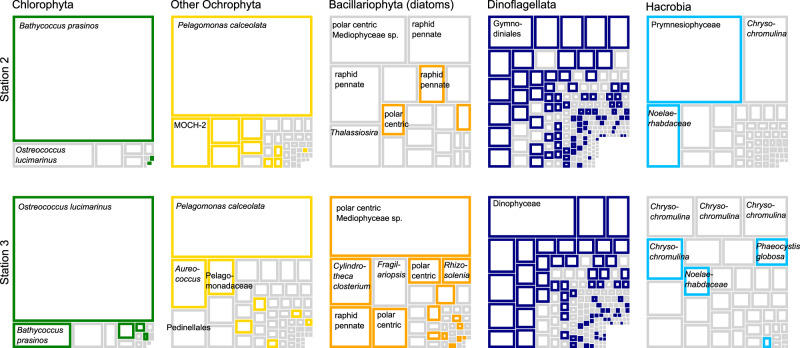
Exported surface phytoplankton ASVs and their relative read abundance in surface seawater at stations 2 and 3, represented by relative box sizes. Relative read abundances of ASVs in each phytoplankton functional group were normalized to the total reads detected of the functional group. Surface ASVs that were also detected in sediment trap samples are represented by colored squares and ASVs that did not appear to be exported are outlined in gray. Taxonomic identities of the most abundant ASVs in each group are labeled at the highest level of classification possible for each sequence variant.

Similarly small proportions of surface phytoplankton diversity (i.e. distinct ASVs) were detected in sinking particles at both the oligotrophic Station 2 (24%) and the CA Current Station 3 (26%) (Fig. 3). However, the relative read abundance of these exported ASVs in the surface differed substantially across locations and among phytoplankton functional types. Exported ASVs at Station 2 comprised a large proportion of surface ochrophyte reads (89%) and hacrobia reads (group including cryptomonads and haptophytes) (49%). Only a small proportion (10%) of surface diatom reads belonged to exported ASVs. The opposite pattern was observed at Station 3, where exported diatom ASVs represented 78% of relative diatom read abundance in the surface and exported ochrophyte and hacrobia ASVs were reduced (58% and 15%, respectively) compared with Station 2. The relative proportion of exported dinoflagellate (Station 2: 67%, Station 3: 78%) and chlorophyte (Station 2: 83%, Station 3: 92%) ASVs throughout the surface mixed-layer was high, irrespective of the ecosystem. The relative read abundance of exported taxa detected between the two trap deployments at Station 3 varied <9% (Supplementary Fig. 4a), suggesting that our samples adequately represented the sinking particles in that ecosystem.

### Identification of taxa exported within small particle size classes

**Fig. 4:**
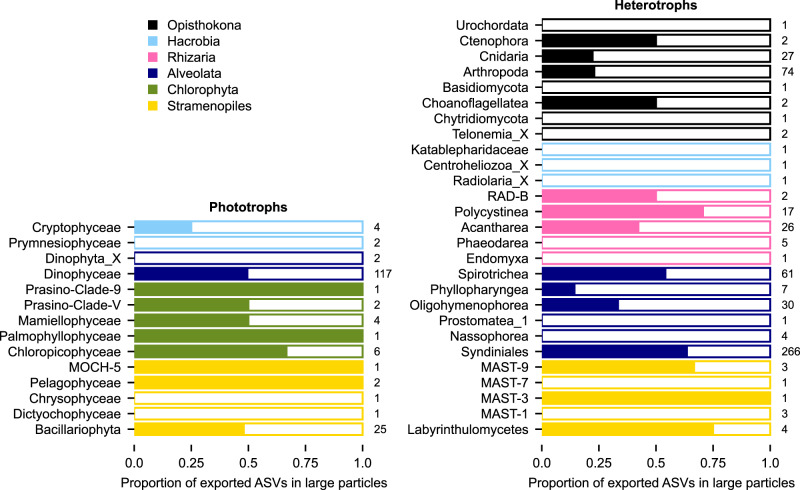
Proportion of exported phytoplankton and heterotroph ASVs present in large, individually isolated particles. Colors signify functional groups and number indicate the total number of ASVs detected in the bulk trap samples.

At Station 3, 49% of both phytoplankton and heterotrophic ASVs detected in sediment traps were present in large particles (Fig. 4), suggesting an equal but distinct role of large and small particles in exporting diversity. This equal partitioning of ASV diversity between large and small particles held true across taxonomic categories except for Chlorophyta, Hacrobia, and Opisthokonta. A greater proportion of exported Chlorophyta diversity (64%) was identified in large particles, whereas exported Hacrobia and Opisthokona ASVs were rarely identified in large particles (23% and 10% of ASVs, respectively). This partitioning was similarly identified in communities from both trap deployments at Station 3 (Supplementary Fig. 4b).

The diatom (Bacillariophyta) ASVs detected in the two sinking size fractions were composed of distinct genera with notable differences in size (Supplementary Table 3) [42]. Specifically, the ASVs of diatom genera found only in the small sinking particle size class were among the largest known diatom cells (*Rhizosolenia*, *Proboscia*, *Coscinodiscus*), whose lengths are typically 0.5 mm to 1 mm. *Bacteriastrum*, whose ASVs were also identified in the small size fraction, are not large in cell size (20–60 µm) but typically form long, spinose chains. In contrast, exported diatom ASVs identified in the large particles included some of the smallest known diatom cells (*Minidiscus*, ~4 μm) and lightly silicified (*Cylindrotheca*, *Guinardia*) diatom genera.

### Differences in 18 S rRNA gene community composition among particle and sample types

**Fig. 5:**
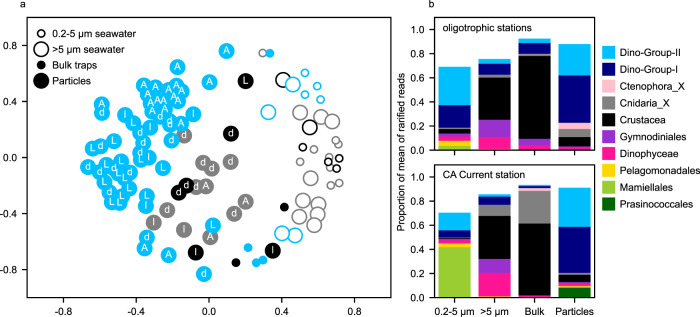
Comparison of 18 S rRNA gene communities detected in different samples types and across locations. **a** Multidimensional scaling ordination of Bray-Curtis dissimilarities of rarified read counts. Samples were collected at the oligotrophic Stations 1 (gray) and 2 (black), and at Station 3 (blue) the California Current. Letters indicate individual particle type if applicable: A = aggregate, L = large loose fecal pellet, d = dense detritus, l = long fecal pellet. **b** Relative composition of rarified read counts grouped by taxonomic Class. Bars represent the mean of all samples within a sample type at the oligotrophic stations (upper) and in the CA Current (lower). Classes composing <1% of the dataset are not plotted.

Samples with similar 18 S rRNA gene community compositions were identified based on rarified relative 18 S rRNA gene sequence reads, and the largest differences in communities occurred among sample types and between the oligotrophic and coastal ecosystems (Fig. 5a). No difference was detected in particle-associated communities collected by two successive trap deployments at Station 3 (Supplementary Fig. 4c). In all sample types, heterotrophic taxa comprised the majority of the total 18 S rRNA gene sequence reads (80 ± 16% of rarified sequence reads across 113 samples, Fig. 5b, Supplementary Fig. 5). Although phytoplankton comprised a smaller proportion of the sequence reads, these organisms still contributed to differences among sample groups. The 0.2–5 µm seawater samples contained a large proportion of Pelagomonadales at oligotrophic stations and a greater proportion of Mamiellales chlorophytes in the California Current. The particles collected in the California Current contained a greater proportion of Prasinococcales chlorophyte reads.

**Fig. 6:**
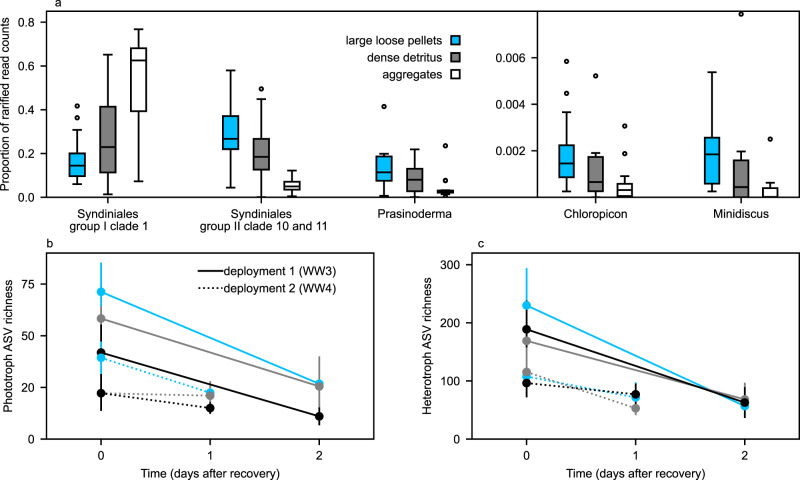
Changes in relative ASV read abundance and diversity among particles at various states of degradation. **a** Taxa with significantly different relative read counts detected in large loose fecal pellets, dense detritus, and aggregates. ASVs were grouped by genus and boxplots indicate relative read abundance of those taxa that differed significantly between large loose pellet and aggregates (*t*-test *p* < 0.001). Middle line indicates the mean, boxes extend to the first quartile, whiskers indicate the range of quartiles, and points indicate data outliers. Boxes represent the distribution of measurements from 16 large loose fecal pellets, 16 dense detritus, and 20 aggregate particles. Changes in **b** phototrophic and **c** heterotrophic ASV richness in particles collected from two separate trap deployments and isolated immediately or after an additional 1 or 2 days. Particle types are indicated by colors as in panel a. Long fecal pellets are not shown because only four particles of this class were isolated in total.

Differences in 18 S rRNA gene community composition also occurred among individually isolated particle classes, which could be assessed at Station 3 due to the large number of particles isolated. The 18 S rRNA gene community composition within aggregates was different from the composition within large loose pellets and dense detritus (PERMANOVA of Bray–Curtis dissimilarities, *p* < 0.001, Fig. 5a). When ASVs were grouped at the genus level, Station 3 large loose pellets only contained significantly more Group-II Clade-10-and-11 Syndiniales alveolate reads, *Chloropicon* and *Prasinoderma* chlorophyte reads, and *Minidiscus* diatom reads (*t*-test, *p* < 0.001; Fig. 6a) compared with aggregates. Aggregates only contained significantly more Group-I Clade-I Syndiniales alveolate reads compared with large loose pellets. Dense detritus particles contained these taxa at abundances intermediate to those of the large loose pellets and aggregates. Total phytoplankton ASV richness decreased in dense detritus and aggregates compared with large loose pellets (ANOVA, *p* < 0.05), and was also associated with the amount of time that had elapsed after trap recovery (Fig. 6b, c). When samples were allowed to degrade an additional one or 2 days at 4 °C, phytoplankton ASV richness was indistinguishable among particle types (ANOVA, *p* > 0.05). By comparison, heterotroph ASV richness did not differ among particle types (ANOVA, *p* > 0.05) and continued to decrease similarly among particles incubated for an additional 1 or 2 days before isolation.

### 18 S rRNA gene taxa most affected by particle degradation

Particle degradation affected the detection of 18 S rRNA gene sequences from various taxa in different ways (Supplementary Fig. 6). Among phytoplankton ASVs, dinoflagellate sequences were the most affected by apparent particle degradation, with a loss of up to 82 ± 31% of ASVs after incubating on the ship. Diatom (Bacillariophyta) sequences were not degraded as rapidly, with ASV richness reduced by 55 ± 40% 2 days after collection. In contrast, ASV richness of Chloropicophyceae chlorophytes did not appear to be affected by particle degradation. ASV richness was reduced in all heterotrophic taxa grouped by Class after incubation on the ship. The ASV diversity detected in bulk trap samples also differed depending on whether collection tubes carried preservative or not (Supplementary Fig. 7), though the direction of this change was not consistent among trap deployments and varied among taxonomic groups.

## Discussion

**Fig. 7:**
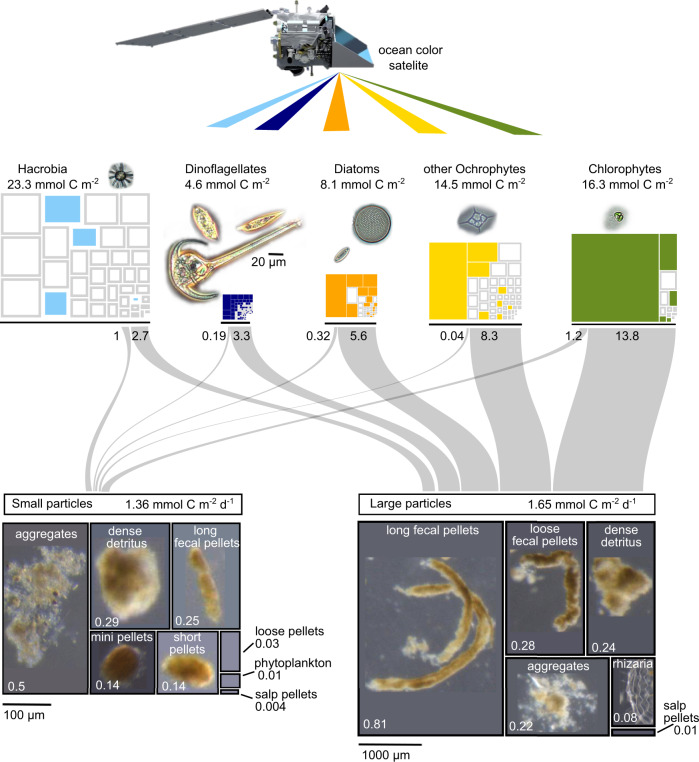
Mechanisms of phytoplankton POC export and particle flux at Station 3 in the California Current between 12–14 February 2017. Treemaps of phytoplankton functional groups are scaled to their biomasses (mmol C m^−2^) estimated by HPLC and integrated through the depth of the mixed layer. ASVs within each functional group were scaled to their relative read abundance in the surface. Colored ASV boxes indicate which taxa were exported. The sankey diagram link width indicates the quantity (mmol C m^−2^) of exported surface phytoplankton biomass packaged in small (<300 µm) versus large particles. The quantity of total carbon exported by various particle types in each size class is represented by the relative box size of the lower treemaps containing example micrograph images, as reported in [28]. The modeled POC flux is labeled on each particle type (mmol C m^−2^ d^−1^). Scale bars for each category of micrograph (phytoplankton, small particles, large particles) are indicated. Micrographs of surface phytoplankton provided by Sunčica Bosak.

Resolving export from the surface ocean at the scale of individual particles enabled us to identify changes in the ecological mechanisms of export between two environments and among functionally distinct taxa. This analysis provided an indication of the taxa responsible for modulating variation in flux across these ecosystems (in this case diatoms, other Ochrophytes, and Hacrobia). The most influential determinant of how a taxon would be exported was the particle size in which it was packaged, which is likely controlled by food web interactions (see below). Importantly, particle degradation had a rapid and measurable impact on detectable diversity within sinking particles, providing a marker of carbon remineralization processes but also affecting how these data can be interpreted. Below we discuss in more detail the ecological significance of these findings and how they might be integrated into quantitative, mechanistic models of the biological carbon pump (Fig. 7).

In both ecosystems (oligotrophic open ocean and mesotrophic coastal), about one quarter of the phytoplankton diversity detected in the surface mixed layer was also detected in sinking particles. Although these exported taxa represented a small fraction of the surface diversity, in many cases they dominated the surface community based on the relative read abundance. Exported Hacrobia and Ochrophyta taxa comprised a relatively high proportion of the reads of each functional type in subtropical surface waters, but only a small proportion of relative surface reads in the CA Current, suggesting a change in their ecological role in the biological pump across these ecosystems. In contrast, exported diatom taxa had low relative read abundance in the subtropical waters and high relative read abundances in the CA Current, where diatoms are well known drivers of the carbon cycle. Valencia et al. [23] also studied this region of the CA Current and, similarly, found that only a small fraction of the surface 18 S rRNA gene diversity was represented in sediment trap collected particles and that diatom sequences were a key component of export. Interestingly, in our study, exported dinoflagellate and chlorophyte reads were relatively abundant in surface waters in both ecosystem types, suggesting that the ecological role of these taxa in the biological pump may be more consistent across the ecosystems.

Relative sequence read abundance can be used as a proxy for relative biomass [10], while acknowledging that this approach has certain limitations and biases. For some taxa (e.g. diatoms), read abundance is a reasonable proxy for relative biomass, while for other taxa (e.g. dinoflagellates) this relationship is less consistent due to large variations in genome size and gene copy numbers among genera [43]. We assessed the relative read abundances within each separate functional group and scaled them to the estimated POC biomass based on HPLC measurements integrated through the depth of the mixed layer, thus translating these data into units that can be incorporated into biological carbon pump models (Fig. 7, upper treemaps in units of mmol C m^−2^).

The export mechanisms of taxa in the California Current appeared to be differentiated by the size of the particle in which they were packaged. About half of the exported phytoplankton and heterotroph diversity was present only in small particles, or those not specifically isolated from gel layers, while the other half was identified in larger individually isolated particles (>300 µm), with some variation among taxonomic groups. This partitioning of sequence diversity pointed to possible mechanisms for their presence in the trap samples. The heterotrophic taxa within these two size fractions could have been transported from the surface or colonized the particles at depth. For example, Opisthokont sequences were primarily detected in the small size fraction, or else those particles not specifically isolated from the gel layers. This supports our interpretation that many of these taxa entered the trap as “swimmer” contaminants that were not targeted for isolation. Unlike a previous study conducted in this region of the CA Current [16], Rhizaria were only a small component of the sequences present in sinking particles and comprised <1% of our dataset. Differences between our data and those of Gutierrez-Rodriguez et al. [16] could be caused by seasonal differences, suggesting that seasonally resolved observations would likely identify important changes in the ecological drivers of export. The diversity of exported taxa detected in surface waters did not fully represent the diversity of taxa detected in the traps, likely due to the different time and space scales over which these sample types integrate.

More interesting was the detection of small sized phytoplankton taxa (chlorophytes, uncultured ochrophyte MOCH-5, pelagophytes) in large particles and the detection of large sized and biomineralized cells in the small particle size fraction. Importantly, this same result was observed within a single taxonomic group, diatoms, indicating that this pattern was not caused by biases in detection among taxonomic lineages. Large diatom genera (*Coscinodiscus*, *Rhizosolenia*, *Proboscia*), which were only detected in small particles, can sink >10 m d^−1^ as individual cells [44], and presumably faster if incorporated into a small aggregate. Previous studies have demonstrated the ability of these large diatoms to sink through the water column [45–48], and our results bolster these earlier conclusions that these large cells can be an important component of the vertical flux. Small diatom cells, which were detected in the largest particles, generally sink <1 m d^−1^ and are unlikely to escape the surface mixed layer unless incorporated into a larger particle, such as a zooplankton fecal pellet [49]. In the California Current, the export of these small cells was likely enhanced by filter feeding zooplankton, like pyrosomes and pteropods, which produce the large loose fecal pellets in which these small cells were packaged. In this way, the presence of specific grazers plays a key role in determining which phytoplankton taxa can contribute to export.

We used this size partitioning to estimate the relative contribution of exported surface phytoplankton in each sinking size fraction (Fig. 7, sankey diagram in units of mmol C m^−2^). From a modeling perspective, the size fraction in which POC is exported affects the relative magnitude of POC flux, the sinking speed, and the rate of POC attenuation with depth. These size fractions are comprised of distinct particle types with different ecological origins (Fig. 7, lower treemaps), which may explain why they package different phytoplankton taxa.

As reported previously [14, 23], sample degradation plays an important role in the detectable diversity of organisms present in aging particles and must be considered when interpreting these data. Differences in community compositions among large loose fecal pellets, dense detritus, and aggregates in the California Current were caused by a sequential reduction in the relative abundance of phytoplankton. Dense detritus and aggregates were probably degraded material originating from fecal pellets, such as the large loose pellets, rather than from a distinct ecological mechanism. Similar evidence of shared particle sources was identified in the Sargasso Sea, where community compositions of different particle types were indistinguishable, indicating a common zooplankton source [12]. In the present study, incubating particles for an additional day or two after collection confirmed that a loss in total 18 S rRNA gene sequence diversity was a symptom of particle degradation and was not uniform across all taxa. Although diversity of all classes of heterotrophs decreased as particles aged, an increase in the relative abundance of certain taxa (e.g. group 1 Syndiniales) suggested that certain microbes may have been growing in the particles. For phytoplankton, the taxa-specific losses in diversity as particles age affects our ability to identify all taxa that contribute to export. While dinoflagellate and diatom sequences showed marked degradation, chlorophyte diversity was essentially unaffected by particle age. Differential DNA preservation was also detected by Valencia et al. [23], who identified a likely difference between the preservation of an organism’s nucleic acids versus its organic carbon within sinking particles. The effects of degradation on diversity within bulk-collected particles were less consistent, similar to a variety of community composition changes observed in previous studies that compared “live” versus preserved trap material [13, 14, 16]. Many of these previous studies have used this treatment comparison to identify evidence of microbial and heterotrophic growth on sinking particles, though various particle fixation and processing strategies have been used, which may have differing effects on the relative community compositions detected. Besides standardizing fixation and processing strategies (e.g. [50]), the rates at which genetic signatures are lost from sinking particle samples must be constrained to better account for biases in detection among different taxa.

The quantity of POC flux associated with each export mechanism (particle size, particle type) identified in this study was previously reported using an image-based modeling approach [28]. Linking these POC flux mechanisms with specific phytoplankton taxa provides the preliminary framework (Fig. 7) needed to develop advanced models of the biological carbon pump. This genetic framework identifies taxa that did not appear to be incorporated into sinking particles, and can identify the specific particle types or sizes into which exported taxa are packaged. These particle types can be modeled with different physical properties such as excess densities, remineralization rate constants, and sinking velocities [51, 52]. These properties then drive the depth of remineralization and energy flow back to microbial communities and to higher trophic level organisms in different size classes. The observations reported in this study thus begin to bridge the gap between the surface phytoplankton community and the parameters that are typically required to tune global scale, biogeochemical models of the biological pump. More work is needed to better constrain the effects of degradation on the detection of genetic signatures. In addition, observations are needed from more locations to identify generalizable ecological linkages among phytoplankton taxa and their export mechanisms. Ultimately, optical signatures that can be observed from the next generation of ocean color satellites could be used to identify surface phytoplankton communities and their associated export processes to provide synoptic information about the biological carbon pump and generate realistically constrained models of the ocean’s carbon cycle [53].

## Supplementary information

Supplemental Material
