# Supplementary material for: Tracing the path of carbon export in the ocean though DNA sequencing of individual sinking particles
Source: ISME J. 2022 Apr 20;16(8):1896–906. doi: 10.1038/s41396-022-01239-2 (PMC9296562; doi:10.1038/s41396-022-01239-2)
Supplement: Supplementary file 1 — Supplemental Material [file 41396_2022_1239_MOESM1_ESM.pdf]

Supplemental Methods

Water column sampling and water property measurements

Vertical profiles of physical and biogeochemical properties of the water column were collected during CTD casts at the three sampling stations (Supplemental Fig. 1). The CTD rosette was equipped with a Sea-Bird Electronics, Inc., SBE 911plus CTD and SBE-42 collecting oxygen data. A Sea-Bird Electronics FLNTU, measuring Chlorophyll Fluorescence (*Chl* F) and backscattering, was mounted on the bottom of the frame, in proximity of Sea-Bird Electronics C-star transmissometer. Underwater Photosynthetically Active Radiation (PAR) was measured using a QSP2300 sensor. We applied several post-processing steps on optical datasets (*Chl* F and attenuation from C-star). Factory calibration was applied to the instrument output to convert the values to [mg m^{-3}] and deep offset (calculated as an average FLNTU *Chl* F channel output at 400 m) was removed from the data. Resulting surface values agreed well with the night values of HPLC based total chlorophyll a (see below). The c-star instrument that was used initially during the cruise, experienced failure while profiling at station 1, resulting in lack of attenuation data for that station. The attenuation (c_p) was calculated from c-star data collected at Stations 2 and 3 and was corrected in a similar fashion to the data collected from *Chl* F instrument (all profiles were intercalibrated at 400 m depth). Particulate organic carbon (POC) profiles were calculated from the attenuation (c_p) using the c_p:POC relationship developed by [1], where POC [mg m^{-3}] = 387.9± 13.2 [mg m^{-2}] * c_p [m^{-1}].

Seawater samples were collected from just below the surface, at the depth of the chlorophyll maximum, and at the bottom of the mixed layer as defined by the depth of

the pycnocline using the CTD Niskin bottles. For pigment analysis, 4 L triplicate seawater samples were filtered onto glass fiber filters, placed in aluminum foil pouches, and immediately stored in liquid nitrogen. Samples were kept at -80ºC for long-term storage. Phytoplankton pigments were determined using high performance liquid chromatography (HPLC) following the procedures of [2] and further described in [3]. Phytoplankton composition for each sample was determined using the EXCEL-based program CHEMical Taxonomy or CHEMTAX program [4], which interprets the ratio of marker pigments to Chl*a* (Pig/Chl*a*) to quantify the abundance of taxonomic groups relative to total Chl*a* biomass. The initial pigment ratio matrix was set using ratios defined in [5] and [6]. The configuration parameters followed those defined in [7], except for 'elements varied' (the number of pigments in the analysis) which was adjusted to 13. Carbon biomass was computed using C/Chla ratios reported in [5]. Pigment-based estimates of POC were assigned to diatoms, dinoflagellates, coccolithophores, cryptophytes, pelagophytes, and green algae, which were used to compare with functional groups identified by DNA sequencing. To compare functional groups resolved by DNA sequences with those of pigment-based estimates of POC, we assumed that the pelagophyte pigment category was representative of ochrophytes and that coccolithophore and cryptophyte pigment categories were representative of hacrobia.

For DNA sampling, water from the three depths at stations 2 and 3 were in some cases combined into a "composite" sample, since the upper layer of the water column was well mixed (see Supplemental Table 1). Either 8 L (stations 1 and 2) or 4 L (station 3) of seawater was filtered in series through a 5 µm and 0.2 µm 47 mm polycarbonate

filters (Whatman, Maidstone, UK) under low vacuum. Filters for DNA were frozen in liquid nitrogen and stored at -80°C.

PCR amplification of 18S rRNA sequences

Approximately 420 bp of the V4 region of 18S rRNA gene sequences in trap-collected particles was amplified by PCR using forward primer TAReuk454FWD1 [8] that included the CS1 tag (Fluidym, San Francisco, CA, USA) on the 5' end (5'-[ACACTGACGACATGGTTCTACA] CCAGCASCYGCGGTAATTCC -3'). The reverse primer TAReukREV3_modified [9] included the CS2 tag on the 5' end (5'-[TACGGTAGCAGAGACTTGGTCT]ACTTTCGTTCTTGATYRATGA - 3'). Triplicate PCR reactions contained 0.3 mM dNTPs, 0.4 μM forward and reverse primers, 5 μL of 5x buffer (Roche, Basel, Switzerland), 0.5 U of polymerase (Kapa HiFi Hotstart PCR kit) and DNA extracted from individual particles (5 μL) or from bulk collected particles (2 μL) in a 25 μL reaction volume. Reactions were incubated at 98°C for 5 min followed by 30-35 cycles at 98°C (30 s), 62°C (45 s), and 72°C (1 min) followed by a final incubation at 72°C for 10 min. Triplicate reactions were combined and purified with 1:1.4 ratio of pcr product to Axyprep PCR clean up kit magnetic beads (Corning, USA). Indexes were added to the amplicons of each sample through an additional short amplification step using a forward primer that included the p5 adaptor (Illumina, USA), an 8 nucleotide barcoding index, and the CS1 tag (5'-AATGATACGGCGACCACCGAGATCTACAC[index] ACACTGACGACATGGTTCTACA-3'). The reverse primer included the p7 adaptor (Illumina), an 8 nucleotide barcoding index, and the CS2 tag (5'-

CAAGCAGAAGACGGCATACGAGAT[index] TACGGTAGCAGAGACTTGGTCT-3'). PCR reactions included 0.4 μM forward and reverse primers, 0.3 mM dNTPs, 4 μL of 5x buffer (Roche), 2 ng of purified PCR product, and 0.4 U of polymerase (Kapa HiFi Hotstart PCR kit) in a 20 μL volume. Reactions were incubated at 98°C for 3 min followed by 7 cycles of 98°C (15 s), 60°C (30 s), and 72°C (1 min) followed by an incubation at 72°C for 3 min. PCR products were purified as before (Axygen PCR clean up kit) and quantified using the Quant-iT PicoGreen dsDNA Assay Kit (Invitrogen, Carlsbad, CA USA). A total of 81 purified, barcoded PCR reactions were combined in equal concentration and sequenced at the Michigan State University Genomics Core Facility using an Illumina MiSeq v2 Standard flow cell in a 2x250 bp paired end format.

DNA from surface seawater samples was extracted with phenol:chlorophorm:isoamyl alcohol solvents (n = 40) as previously described [10]. DNA samples were sent for 18S rRNA gene library preparation and sequencing to Molecular Research MrDNA (Shallowater, TX, USA). The V4 region of the 18S rRNA was amplified by the sequencing facility using the TAReuk454FWD1 and TAReukREV3 modified primers [8, 9] and HotStarTaq Plus Master Mix Kit (Qiagen) under the following conditions: 94°C for 3 mi, followed by 28 cycles of 94°C for 30 seconds, 53°C for 40 seconds, and 72°C for 1 minute, after which a final elongation step at 72°C for 5 minutes was performed. Sequencing of 2x300 bp paired-end read was performed on an Illumina MiSeq platform. Sequencing of one seawater sample (Station 2, 98 m, >5 μm size fraction) was not successful.

Description of 18S rRNA sequence data processing using Qiime 2

Sequence data generated from the sediment trap and seawater samples were each imported into Qiime2 and demultiplexed if needed. DADA2 was used to trim off primers, trim low-quality sequence regions, join paired-end reads, filter out low quality sequences, and identify amplicon sequence variants (ASVs) in a feature table with the number of reads detected for each ASV (qiime dada2 denoise-paired). Sequence quality over the length of the amplicons was manually assessed in QiimeView using the "qiime demux summarize" function (--o-visualization). The average quality score of sequences generated from sediment trap particles remained high for the entire 250 bp length while the average quality of sequences generated from surface samples decrease (median quality <30) at the 3' ends of both forward and reverse 300 bp reads and were therefore trimmed to 260 bp using DADA2. The sequences and ASV feature tables from both the seawater and sediment trap samples were then merged into combined data files (qiime feature-table merge, qiime feature-table merge-seqs). ASVs were associated with taxonomic identities by comparison to the PR2 database [11](v 4.12.0, downloaded 24 January 2020). A naive bayes classifier was trained on the PR2 dataset by extracting reads with the TAReuk454FWD1 and TAReukREV3_modified primer sequences (qiime feature-classifier extract-reads, qiime feature-classifier fit-classifier-naive-bayes). The classifier was then used to assign taxonomic identities to the combined seawater and trap DNA sequences (qiime feature-classifier classify-sklearn).

Assessing methodological biases affecting comparisons among sample types

The total number of reads sequenced among samples varied among the different sequencing runs for seawater samples (68 807 ± 20 457 reads, n=37) versus trap particles (102 963 ± 20 028 reads, n=81), but all samples were sequenced deeply enough to resolve the ASV richness of the phytoplankton sequence community within each sample (Supplemental Fig. 2); ASV richness did not increase substantially after approximately 10,000 reads. Instead, differences in shared versus unique phytoplankton ASVs among sample types reflected inherent differences in how each type subsampled the environment with differing probabilities of detecting a particular organism (Supplemental Fig. 3). If no methodological bias existed, all phytoplankton ASVs detected in individual particles would be identified in the bulk trap particles, and all phytoplankton ASVs detected in bulk trap particles would be identified in the surface seawater. Instead, a fraction of the phytoplankton ASVs in each sample type were unique or shared with one or both of the other sample types (Supplemental Fig. 3a).

When a greater number of individual particles were sampled from the gel layers, additional phytoplankton ASVs were detected but were mostly unique to those individual particles (Supplemental Fig. 3b). The detection of unique ASVs within individual particles seemed to be caused by the higher sequencing effort applied to these samples compared to the other sample types. Rare, low-abundance sequence were more likely be detected in 100,000 reads sequenced from an individual particle than in the same number of reads from a heterogeneous bulk trap sample. Only small changes in the number of phytoplankton ASVs shared across sample types were observed when more individual particles were sampled, and this had varying effects on our assessment of

phytoplankton export mechanisms. The total phytoplankton sequence community from a random subset (n=6, 10, 20, 30, 40, or 50) of particles collected at Station 3 was sampled 100 times to simulate the effects of particle sampling size on our analyses. Particle sample size had a relatively small effect on the percent of surface ASVs detected in trap material (Supplemental Fig. 3c). An exponential decay function fitted to the simulated data ($y = 0.15 \times [1\text{-}e^{-0.03x}]+0.14$) indicated that infinite particle sampling would theoretically identify 29% of the surface ASVs in the trap material at station 3. In contrast, the number of sampled particles had a large effect on our interpretation of the size fractions in which phytoplankton taxa were packaged, inferred from the total bulk trap phytoplankton ASVs also detected in individually isolated sinking particles (Supplemental Fig. 3d). Simulated subsampling of station 3 particle data indicated that too few particles were sampled at station 2 to accurately estimate the size fraction in which taxa was exported, but the 57 particles sampled at station 3 were adequate for this analysis. An exponential decay function fitted to the simulated subsamples ($y = 0.37 \times [1\text{-}e^{-0.05x}]+0.14$) indicated that infinite particle sampling would theoretically detect 51% of exported phytoplankton ASVs in large, individually isolated particles at station 3.

Annotated code used in Qiime2

```
###Qiime2 analysis notes###
#version qiime2-2019.7
############################################################

##Working with FK170124 18S data from MSU sequencing facility (Traps) and MrDNA (surface seawater)
##first import data.  Already demultiplexed and barcodes have been removed.
##Traps still have primers but surface do not.

##################################
##Import and process trap data###
##################################
qiime tools import \
```

```
--type 'SampleData[PairedEndSequencesWithQuality]' \
--input-path
/home/cdurkin/cdurkin_shared/data/Amplicons/FK170124/20180411_18S_PE/20180411_18S_PE_rawre
ads/
--input-format CasavaOneEightSingleLanePerSampleDirFmt \
--output-path demux-paired-end.qza

qiime demux summarize \
--i-data demux-paired-end.qza \
--o-visualization demux.qzv

##use dada2 to quality control the paired end data
##Trim off the primer sequences, and don't trim off any of the 3' end because high quality

qiime dada2 denoise-paired \
--i-demultiplexed-seqs demux-paired-end.qza \
--o-representative-sequences rep-seqs.qza \
--o-table table.qza \
--o-denoising-stats stats.qza
--p-trunc-len-f 250 \
--p-trunc-len-r 250 \
--p-trim-left-f 20 \
--p-trim-left-r 21

qiime feature-table summarize \
  --i-table table.qza \
  --o-visualization table.qzv \
  --m-sample-metadata-file metadata_FK170124.tsv

qiime feature-table tabulate-seqs \
  --i-data rep-seqs.qza \
  --o-visualization rep-seqs.qzv

qiime metadata tabulate \
  --m-input-file stats.qza \
  --o-visualization stats.qzv

########################################
##Import and process Seawater data###
#######################################

qiime tools import \
  --type EMPPairedEndSequences \
  --input-path
/home/cdurkin/cdurkin_shared/data/Amplicons/FK170124/FK170124_18S_seawater/seawater_EMP_pair
edend/\
  --output-path surface-paired-end-sequences.qza

qiime demux emp-paired \
  --m-barcodes-file sample-metadata.tsv \
  --m-barcodes-column BarcodeSequence \
  --p-no-golay-error-correction \
  --i-seqs surface-paired-end-sequences.qza \
  --o-per-sample-sequences surface_demux.qza \
  --o-error-correction-details surface_demux-details.qza
```

```
qiime demux summarize \
  --i-data surface_demux.qza \
  --o-visualization surface_demux.qzv


###Dada2 processing of surface seawater sequences##
###primer has already been removed, trimming lower quality 3' ends to 260##

qiime dada2 denoise-paired \
--i-demultiplexed-seqs surface_demux.qza \
--o-representative-sequences surface_rep-seqs.qza \
--o-table surface_table.qza \
--o-denoising-stats surface_stats.qza \
--p-trunc-len-f 260 \
--p-trunc-len-r 260 \

qiime metadata tabulate \
  --m-input-file stats.qza \
  --o-visualization stats.qzv

###############################################
###Combine data from multiple sequencing runs##
###############################################

qiime feature-table merge \
  --i-tables /home/projects/FK170124/FK170124_18S_surface/surface_table.qza  \
  --i-tables /home/projects/FK170124/FK170124_18S/table.qza \
  --o-merged-table /home/projects/FK170124/FK170124_18S_Traps_CTD/Traps_CTD_table.qza

qiime feature-table merge-seqs \
  --i-data /home/projects/FK170124/FK170124_18S_surface/surface_rep-seqs.qza \
  --i-data /home/projects/FK170124/FK170124_18S/rep-seqs.qza  \
  --o-merged-data Traps_CTD_rep-seqs.qza

qiime feature-table tabulate-seqs \
  --i-data Traps_CTD_rep-seqs.qza \
  --o-visualization Traps_CTD_rep-seqs.qzv

qiime tools extract \
  --input-path /home/projects/FK170124/FK170124_18S_Traps_CTD/Traps_CTD_table.qza \
  --output-path /home/projects/FK170124/FK170124_18S_Traps_CTD/Traps_CTD_Feature_table.qza

biom convert -i feature-table.biom -o Traps_CTD_feature_table --to-tsv

#######################################
#Training PR2 database used to classify 18S rRNA sequences
#Website for PR2 database (v 4.12.0, downloaded 01/24/2020):
#https://github.com/pr2database/pr2database/releases

qiime tools import \
  --type 'FeatureData[Sequence]' \
  --input-path /home/cdurkin/cdurkin_shared/data/Amplicons/PR2/pr2_version_4.12.0_18S_mothur.fasta \
  --output-path /home/cdurkin/cdurkin_shared/data/Amplicons/PR2/pr2_version_4.12.0_18S_mothur.qza

qiime tools import \
  --type 'FeatureData[Taxonomy]' \
```

```
  --input-format HeaderlessTSVTaxonomyFormat \
  --input-path /home/cdurkin/cdurkin_shared/data/Amplicons/PR2/pr2_version_4.12.0_18S_mothur.tax \
  --output-path /home/cdurkin/cdurkin_shared/data/Amplicons/PR2/pr2-ref-taxonomy.qza

qiime feature-classifier extract-reads \
  --i-sequences /home/cdurkin/cdurkin_shared/data/Amplicons/PR2/pr2_version_4.12.0_18S_mothur.qza
\
  --p-f-primer CCAGCASCYGCGGTAATTCC \
  --p-r-primer ACTTTCGTTCTTGATYRATGA \
  --p-min-length 200 \
  --p-max-length 500 \
  --o-reads /home/cdurkin/cdurkin_shared/data/Amplicons/PR2/18Sref-seqsP2.qza

qiime feature-classifier fit-classifier-naive-bayes \
  --i-reference-reads /home/cdurkin/cdurkin_shared/data/Amplicons/PR2/18Sref-seqsP2.qza \
  --i-reference-taxonomy /home/cdurkin/cdurkin_shared/data/Amplicons/PR2/pr2-ref-taxonomy.qza \
  --o-classifier /home/cdurkin/cdurkin_shared/data/Amplicons/PR2/PR2classifier.qza

#########################################
####Classify 18S sequences
qiime feature-classifier classify-sklearn \
  --i-classifier /home/cdurkin/cdurkin_shared/data/Amplicons/PR2/PR2classifier.qza \
  --i-reads Trap_CTD_rep-seqs.qza \
  --o-classification Trap_CTD_taxonomy.qza

qiime metadata tabulate \
  --m-input-file taxonomy.qza \
  --o-visualization taxonomy.qzv


##Rarify the count data in the feature table###
###Rarify to 31993.  This is the sequencing depth of sample 13##
qiime diversity core-metrics \
        --i-table ./Traps_CTD_table.qza \
        --p-sampling-depth 31993 \
        --m-metadata-file ./metadata_FK170124_Traps_CTD.tsv \
        --o-rarefied-table Traps_CTD_31993_rarified_feature_table.qza \
        --o-bray-curtis-distance-matrix Traps_CTD_31993_BC_matrix_rarified_data.qza \
        --o-observed-otus-vector Traps_CTD_31993_obs_OTUS.qza \
        --o-shannon-vector Traps_CTD_31993_shannon_vector.qza \
        --o-evenness-vector Traps_CTD_31993_evenness_vectore.qza \
        --o-jaccard-distance-matrix Traps_CTD_31993_jaccard_matrix.qza \
        --o-jaccard-pcoa-results Traps_CTD_31993_jaccard_pcoa.qza \
        --o-bray-curtis-pcoa-results Traps_CTD_31993_BC_pcoa.qza \
        --o-jaccard-emperor Traps_CTD_31993_jaccard_emperor.qza \
        --o-bray-curtis-emperor Traps_CTD_31993_BC_emperor.qza \
        --output-dir /home/cdurkin/projects/FK170124/FK170124_18S_Traps_CTD/beta_diversity_stats3/

##Export rarified otu table to analyze in python/jupyter notebook

qiime tools extract \
  --input-path Traps_CTD_31993_rarified_feature_table.qza \
  --output-path ../FK170124_18s_Trap_CTD_31993_rarified_FeatureTable

biom convert -i feature-table.biom -o FK170124_18S_Traps_CTD_31993_rarified_feature_table --to-tsv
```

Supplemental Table 1. Depths over which the surface mixed layer was sampled at three stations. Composite depths are marked with asterisk.

Station	Date	Surface (m)	Chl max (m)	ML depth (m)	Additional discrete depths (m)
1	28 Jan 2017	0	115	130	
	29 Jan 2017	0	115	125	
	5 Feb 2017	0	88	128	
2	6 Feb 2017	0	98	128	
	7 Feb 2017	0*	95 *	120 *	
3	12 Feb 2017	0*	31*	42*	
	13 Feb 2017	0 *	24*	30 *	24, 30

Supplemental Table 2. Taxonomic categories from the PR2 database considered to be photosynthetic or have the potential to be photosynthetic and the number of unique ASVs within each taxonomic grouping.

Phytoplankton Taxa	# of
Eukaryota;Alveolata;Dinoflagellata;Dinophyceae	1876
Eukaryota;Alveolata;Dinoflagellata;Dinophyta_X	24
Eukaryota;Alveolata;Dinoflagellata;Ellobiophyceae	1
Eukaryota;Alveolata;Dinoflagellata;Noctilucophyceae	12
Eukaryota;Archaeplastida;Chlorophyta;Chlorodendrophyceae	1
Eukaryota;Archaeplastida;Chlorophyta;Chlorophyceae	3
Eukaryota;Archaeplastida;Chlorophyta;Chlorophyta_X	1
Eukaryota;Archaeplastida;Chlorophyta;Chloropicophyceae	63
Eukaryota;Archaeplastida;Chlorophyta;Mamiellophyceae	38
Eukaryota;Archaeplastida;Chlorophyta;Palmophyllophyceae	30
Eukaryota;Archaeplastida;Chlorophyta;Prasino-Clade-9	17
Eukaryota;Archaeplastida;Chlorophyta;Prasino-Clade-V	3
Eukaryota;Archaeplastida;Chlorophyta;Pyramimonadales	14
Eukaryota;Archaeplastida;Chlorophyta;Ulvophyceae	1
Eukaryota;Excavata;Discoba;Euglenozoa	4
Eukaryota;Hacrobia;Cryptophyta;Cryptophyceae	25
Eukaryota;Hacrobia;Haptophyta;Haptophyta_Clade_HAP2	3
Eukaryota;Hacrobia;Haptophyta;Haptophyta_Clade_HAP3	6
Eukaryota;Hacrobia;Haptophyta;Haptophyta_Clade_HAP4	9

Eukaryota;Hacrobia;Haptophyta;Haptophyta_Clade_HAP5	3
Eukaryota;Hacrobia;Haptophyta;Haptophyta_X	5
Eukaryota;Hacrobia;Haptophyta;Prymnesiophyceae	136
Eukaryota;Rhizaria;Cercozoa;Chlorarachniophyceae	5
Eukaryota;Stramenopiles;Ochrophyta;Bacillariophyta	228
Eukaryota;Stramenopiles;Ochrophyta;Bolidophyceae	17
Eukaryota;Stramenopiles;Ochrophyta;Chrysophyceae	48
Eukaryota;Stramenopiles;Ochrophyta;Dictyochophyceae	50
Eukaryota;Stramenopiles;Ochrophyta;MOCH-2	34
Eukaryota;Stramenopiles;Ochrophyta;MOCH-3	3
Eukaryota;Stramenopiles;Ochrophyta;MOCH-4	2
Eukaryota;Stramenopiles;Ochrophyta;MOCH-5	18
Eukaryota;Stramenopiles;Ochrophyta;Pelagophyceae	39
Eukaryota;Stramenopiles;Ochrophyta;Phaeophyceae	3
Eukaryota;Stramenopiles;Ochrophyta;Raphidophyceae	5

Supplemental Table 3. Exported diatom genera transported in small (<300 μm) or large (> 300 μm) particle size fractions, as inferred by whether ASVs present in bulk sediment trap samples were also present in individually isolated particles. ASVs were grouped by their highest taxonomic classification and are labeled here by genus, if possible. Some genera were identified in both "small" and "large" fractions because distinct ASVs in each size category could not be classified to distinct taxonomic identities.

Present in large particles	Cell size (μm)	Particle size fraction
Cylindrotheca	30-400	large
Minidiscus	2-7	large
Guinardia	100-300	large
Navicula	30 -130	large
Thalassiosira	4-190	small and large
Chaetoceros	3 - 80	small and large
Pseudo-nitzschia	20 - 160	small and large
Polar-centric-Mediophyceae	N/A	small and large
Bacteriastrum	20-60	small
Proboscia	~500-1000	small

Rhizosolenia	~100-1000	small
Coscinodiscus	30-500	small
Raphid-pennate	N/A	small
Bacillariophyta	N/A	small

Supplemental References

1. Graff JR, Nelson NB, Roca-Martí M, Romanelli E, Kramer S, Erickson Z, et al. Reconciliation of total particulate organic carbon and nitrogen measurements determined using contrasting methods in the North Pacific Ocean. submitted.
2. Van Heukelem L, Thomas CS. Computer-assisted high-performance liquid chromatography method development with applications to the isolation and analysis of phytoplankton pigments. *Journal of Chromatography A* 2001; **910**: 31–49.
3. Hooker SB, Heukelem L, Thomas C, Claustre H, Ras J, Barlow R, et al. The Second SeaWiFS HPLC Analysis Round-Robin Experiment (SeaHARRE-2). *NASA Technical Memorandum* 2005; 1–112.
4. Mackey MD, Mackey DJ, Higgins HW, Wright SW. CHEMTAX—a program for estimating class abundances from chemical markers: application to HPLC measurements of phytoplankton. *Marine Ecology Progress Series* 1996; **144**: 265–283.
5. Neeley A, Lomas M, Mannino A, Thomas C, Vandermeulen R. Impact of growth phase, evolution and climate change conditions on the cellular pigment and carbon content of fifty-one phytoplankton isolates. submitted.

6. Higgins HW, Wright SW, Schluter L. Quantitative interpretation of chemotaxonomic pigment data. *Phytoplankton Pigments: Characterization, Chemotaxonomy, and Applications in Oceanography*. 2011. Cambridge University Press, United Kingdom, pp 257–301.
7. Kozlowski WA. Pigment derived phytoplankton composition along the Western Antarctic Peninsula. *Master's thesis (San Diego State Univ, San Diego)* 2008.
8. Stoeck T, Bass D, Nebel M, Christen R, Jones M, Breiner H-W, et al. Multiple marker parallel tag environmental DNA sequencing reveals a highly complex eukaryotic community in marine anoxic water. *Molecular Ecology* 2010; **19**: 21–31.
9. Penna A, Casabianca S, Guerra A, Vernesi C, Scardi M. Analysis of phytoplankton assemblage structure in the Mediterranean Sea based on high-throughput sequencing of partial 18S rRNA sequences. *Marine Genomics* 2017; **36**.
10. Massana R, Murray AE, Preston CM, DeLong EF. Vertical distribution and phylogenetic characterization of marine planktonic Archaea in the Santa Barbara Channel. *Appl Environ Microbiol* 1997; **63**: 50.
11. Guillou L, Bachar D, Audic S, Bass D, Berney C, Bittner L, et al. The Protist Ribosomal Reference database (PR2): a catalog of unicellular eukaryote Small Sub-Unit rRNA sequences with curated taxonomy. *Nucleic Acids Research* 2012; **41**: D597–D604.

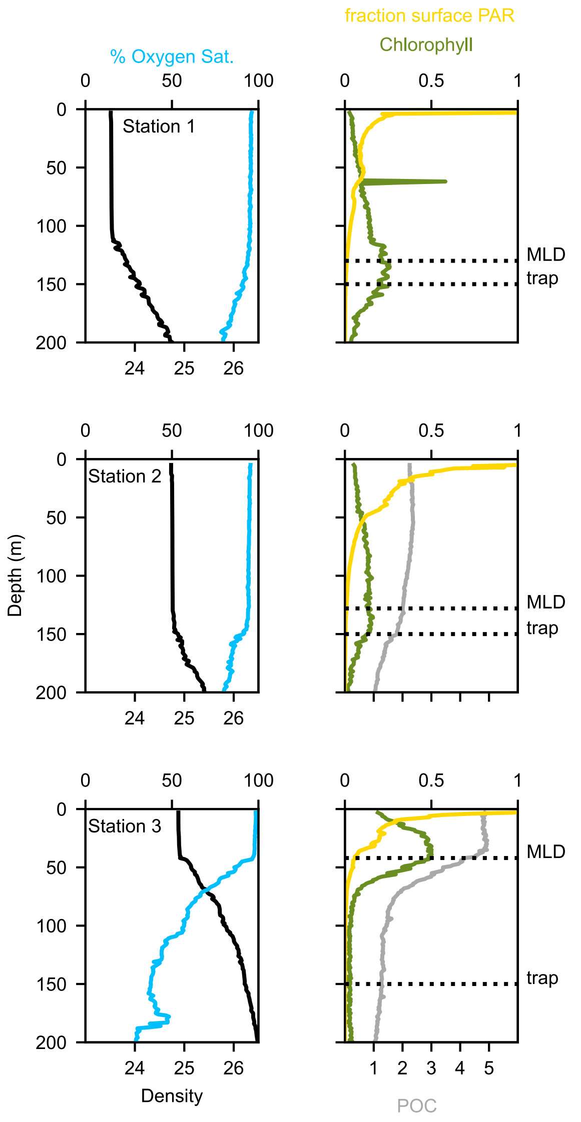

Supplemental Figure 1. Profiles of water column properties as measured by a CTD profiler at stations 1, 2, and 3. Sensors measured % oxygen saturation (blue), density (black, kg m^{-3}), photosynthetically active radiation (PAR, yellow) as a fraction of the surface irradiance, chlorophyll fluorescence (green, mg m^{-3}), and backscatter (c_p) used as a proxy for particulate organic carbon (POC, grey, mmol m^{-3}). Dashed lines indicate the mixed layer depth (MLD) and the sediment trap deployment depth.

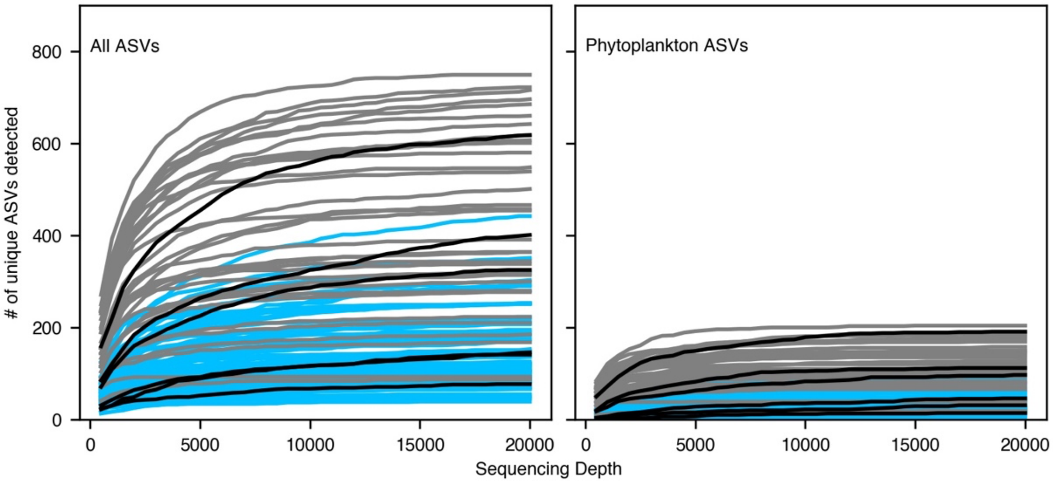

Supplemental Figure 2. Rarifaction curves of all ASVs (left) and phytoplankton-only ASVs (right) detected in all samples, including seawater communities (grey), bulk sediment trap particles (black), and individually isolated particles (blue).

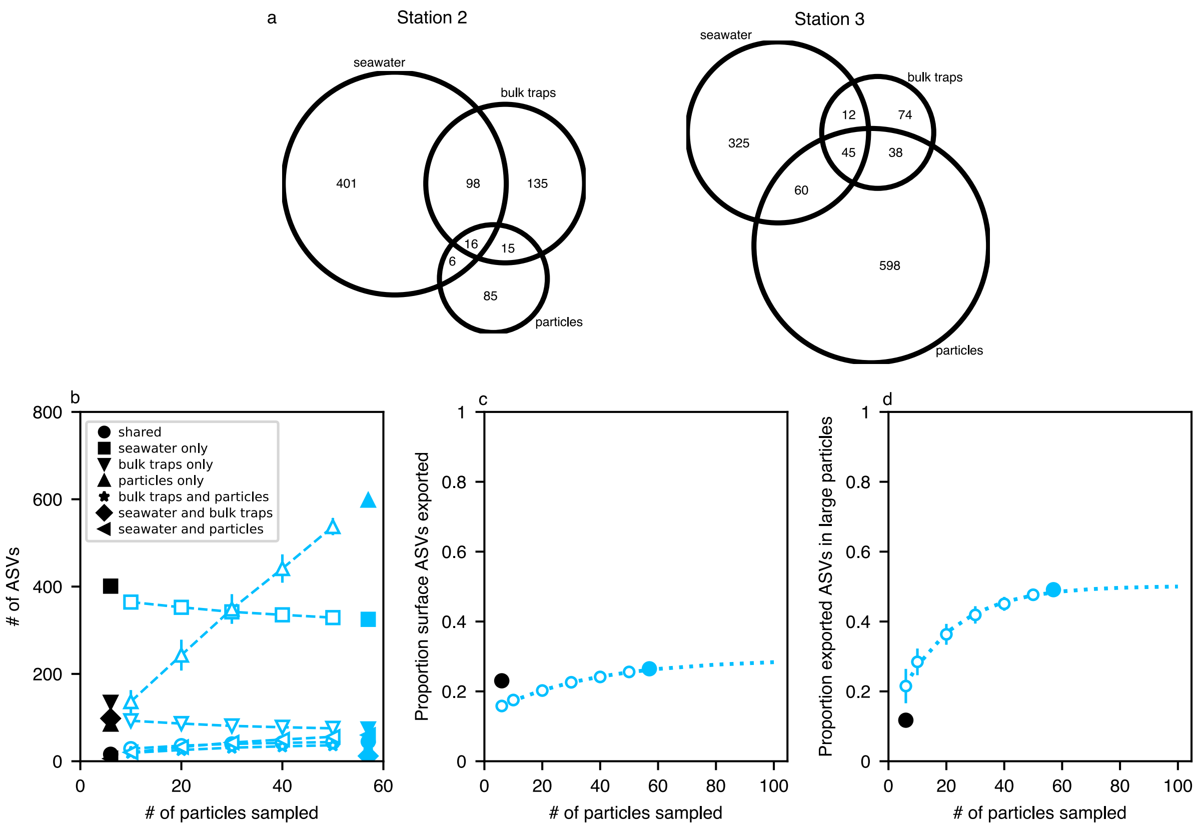

Supplemental Figure 3. a) Number of shared and unique phytoplankton ASVs identified in each sample type at Station 2 and 3. Bulk trap samples were not available from Station 1 for this analysis. b-d) Effect of the number of particles sampled on detection of shared and unique ASVs among sample types. At Station 2, 6 individual particles were sequenced and 2 bulk trap samples (unpreserved brine and RNAlater, black closed symbols). At Station 3, 57 particles and 4 bulk trap samples were sequenced from two consecutive trap deployments (blue closed symbols). Station 3 particles were subsampled to simulate the effect of changing particle sample size (blue open symbols). b) Changes in the number of shared and unique ASVs. c) Effect of the particles sample size on the apparent proportion of surface ASVs exported. Best fit regression was fit to simulated data: $y = 0.15 * (1-e^{-0.03x})+0.14$. d) Effect of the particles sample size on the apparent size fraction in with exported ASVs were packaged. Best fit regression was fit to simulated data: $y = 0.37 * (1-e^{-0.05x})+0.14$.

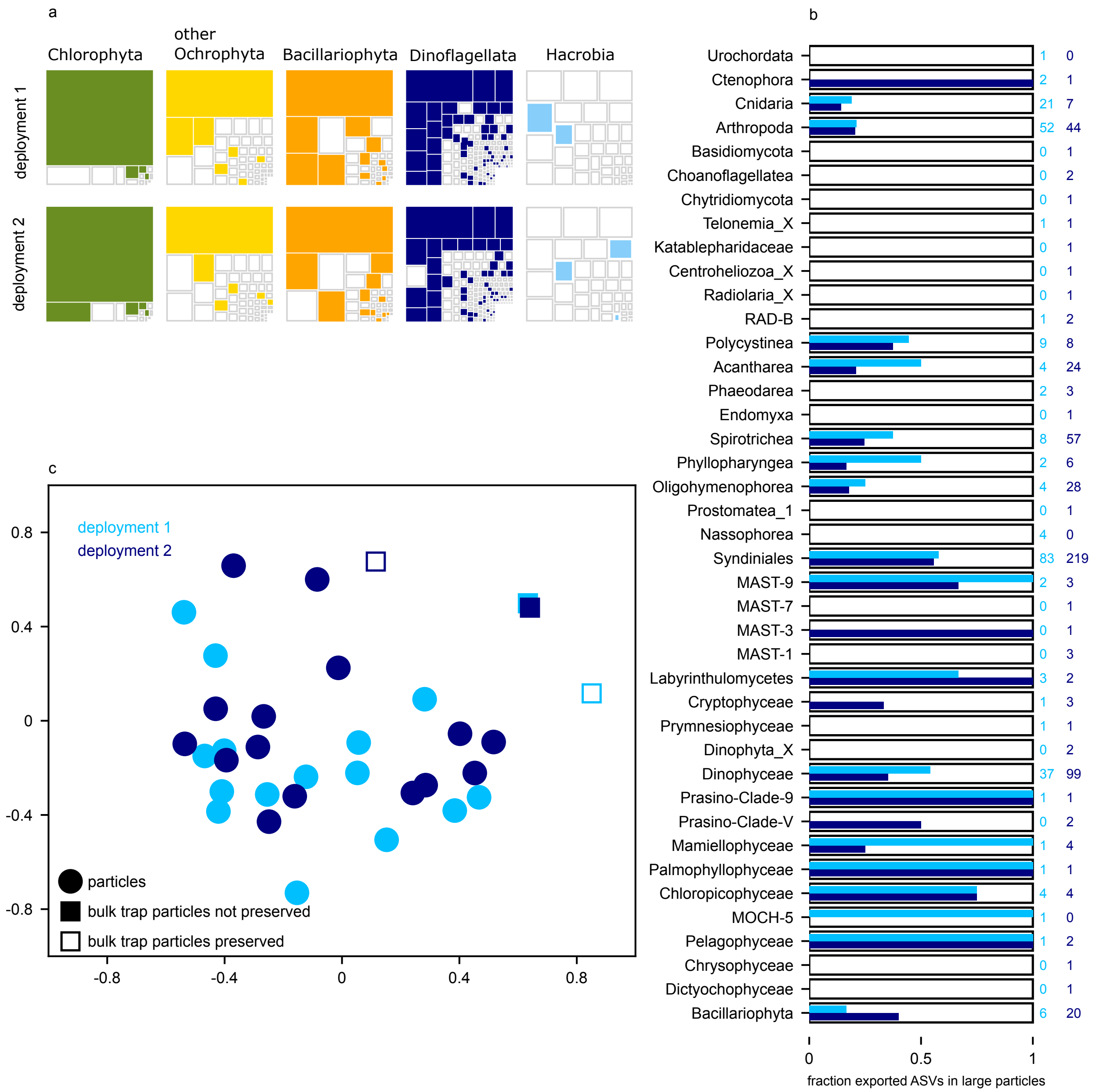

Supplemental Figure 4. Variability in the communities detected between two successive sediment trap deployments at Station 3 (WW3 vs. WW4) to determine the effect of stochastic particle collection on data interpretation. a) Relative read abundance of surface phytoplankton community ASVs associated with major functional groups. Exported ASVs detected in each sediment trap deployment are represented by colored squares. b) The fraction of ASVs detected in large particles in trap deployment 1 (light blue) and deployment 2 (dark blue). Numbers at right indicate the total number if ASVs detected in the bulk trap samples from each deployment. c) Multidimensional scaling analysis of the Bray-Curtis dissimilarities among 18S rRNA communities detected in particles collected during each trap deployment at Station 3.

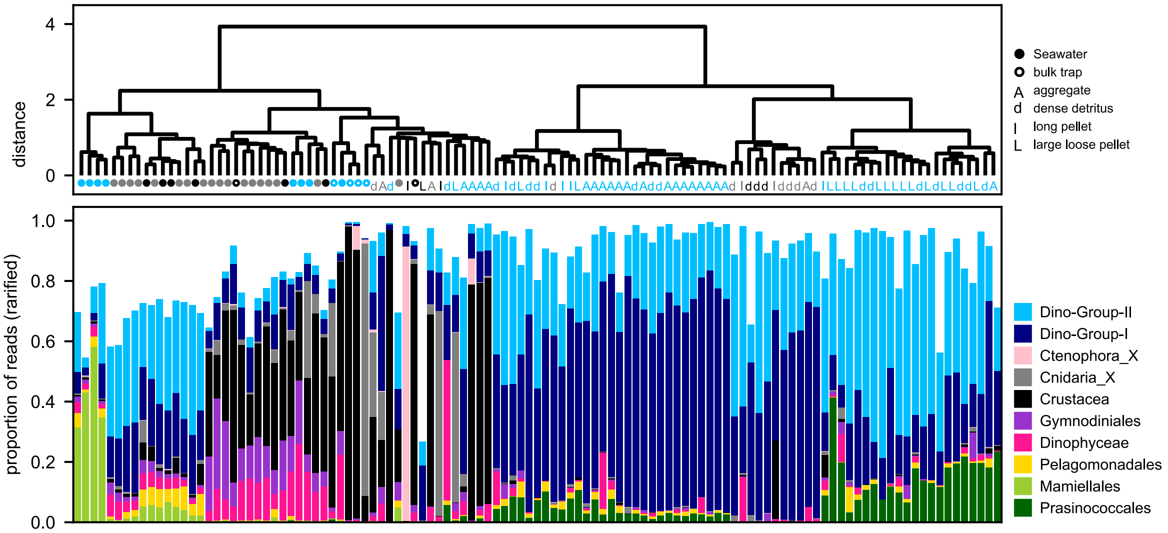

Supplemental Figure 5. Composition of rarified read counts in each sample, grouped by taxonomic Class as a proportion of the total reads. Classes composing less than 1% of the dataset are not plotted. Samples are arranged by similarity of 18S rRNA communities based on Ward hierarchical clustering of Bray-Curtis dissimilarities of rarified read counts (upper panel). Samples were collected at station 1 (grey leaf labels), station 2 (black leaf labels), and station 3 (blue leaf labels).

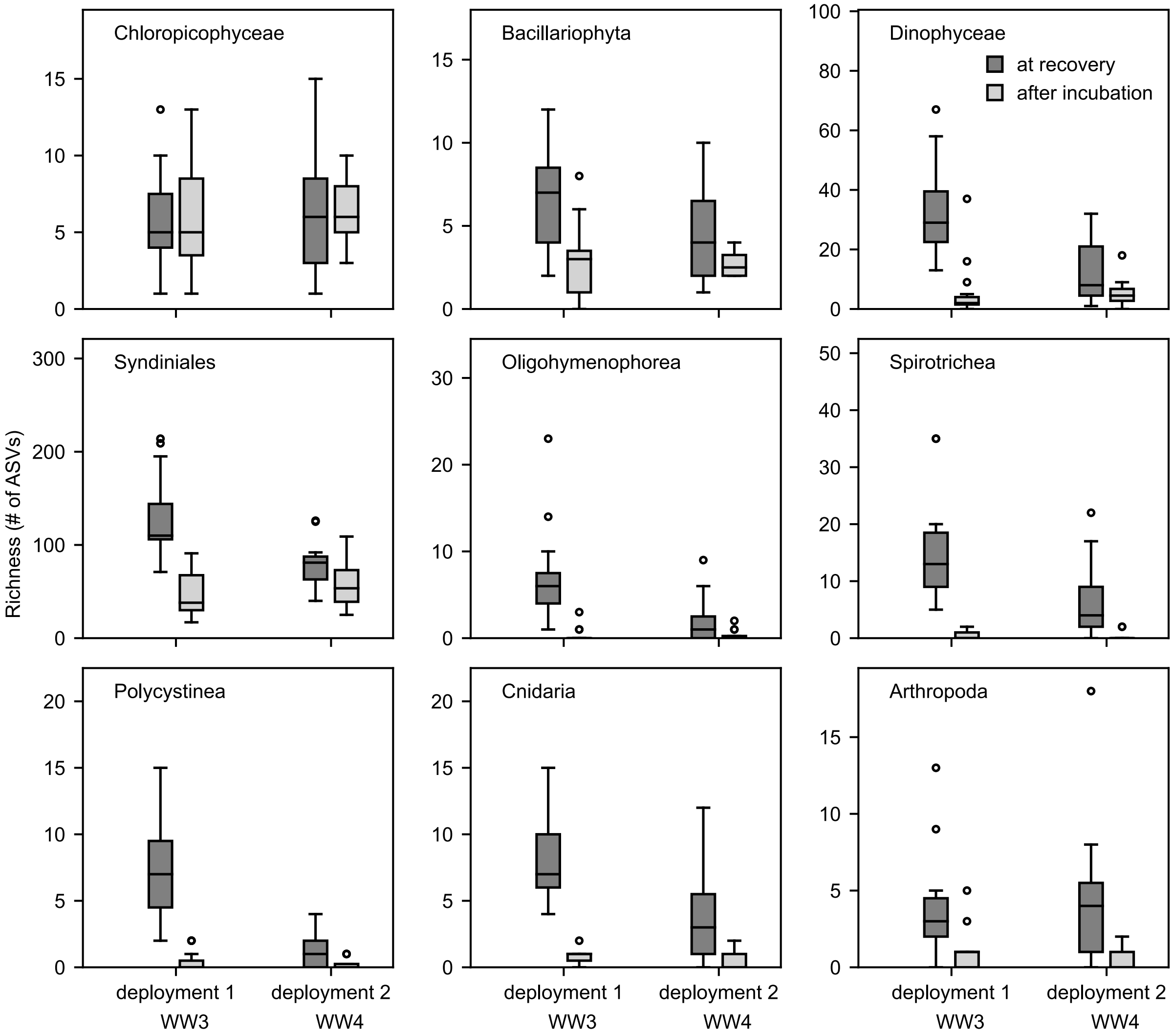

Supplemental Figure 6. Taxa-specific changes in ASV richness detected in individually isolated particles in the California Current. Particles were immediately isolated from two, 1-day trap deployments. Additional particles from these samples were isolated after incubating on the ship for an additional one (deployment 2) or two (deployment 1) days after trap recovery. Taxa were grouped by Class and only those with at least 10 different ASVs detected are shown. Middle line in each box plot indicates the mean, boxes extend to the first quartile, whiskers indicate the range of quartiles, and circles indicate data outliers. Note the scale changes in the y-axes.

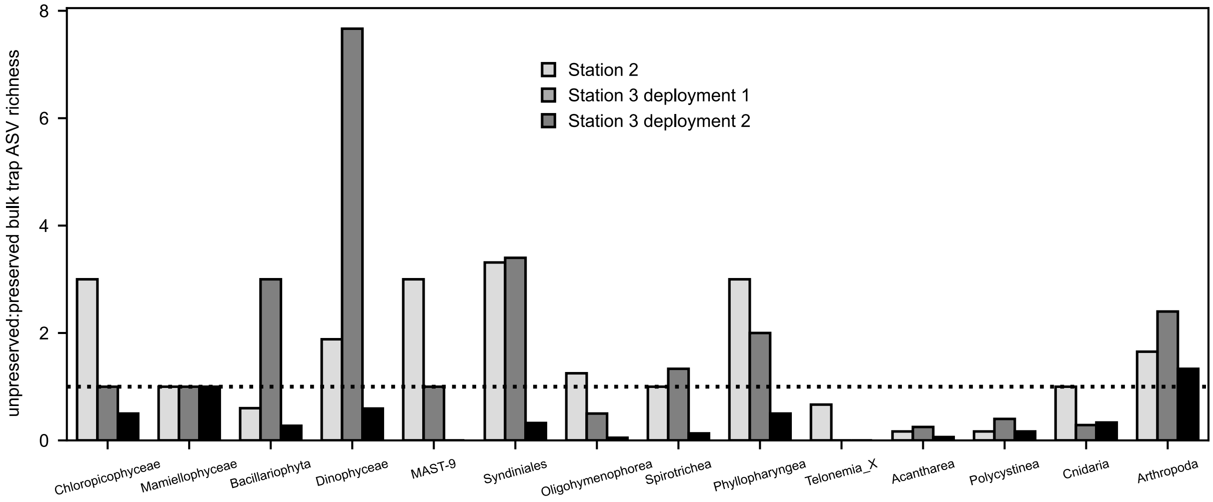

Supplemental Figure 7. Taxa-specific fold change of ASV richness detected in unpreserved bulk-collected sediment trap particles versus those particles preserved in RNAlater from the same deployment. ASVs were grouped by taxonomic Class and only those Classes with at least 10 distinct ASVs are shown. Dashed line indicates no fold change in ASV richness.
